# Regression to the mean explains perception of action consequences

**DOI:** 10.1098/rspb.2025.1715

**Published:** 2025-10-15

**Authors:** Saskia Johnen, Eckart Zimmermann

**Affiliations:** ^1^ Institute for Experimental Psychology, Faculty of Mathematics and Natural Sciences, Heinrich Heine University, Düsseldorf 40225, Germany

**Keywords:** sensory attenuation, sensory enhancement, adaptation to mean sound level

## Abstract

Predictions shape the perceptual consequences of our own actions such that self-generated events appear less intense to us. However, recent studies also reported sensory enhancement of self-produced sounds. Here, we tested whether sensory attenuation and enhancement are signatures of an adaptation to the mean sound statistics. In 330 human participants, we tested the idea that predictions about upcoming sounds shift auditory processing to the average sound context. Participants produced sounds between 40 and 80 decibels (dB) and rated their loudness. Estimates of perceived loudness followed a regression to the mean sound level. The effect was similar for self-produced and passively observed but temporally predictable tones, suggesting predictability alone drives perceptual changes. We then artificially created a new mean sound level by presenting sessions in which subjects mostly (80% of trials) produced either loud (80 dB) or quiet (40 dB) tones. In loud contexts, rarely presented quiet tones were enhanced, and in quiet contexts, loud tones were attenuated. Our results challenge the dominant forward model explanation, which attributes sensory attenuation to predictive suppression of self-generated stimuli, and instead open the door for alternative explanations. Our findings point to regression towards the mean sound level as the most plausible account for predictable sounds.

## 1. Introduction

How predictions shape perception is one of the major threads underlying current perceptual neuroscience. Several models compete to explain the impact of prediction on experience. The testbed of these theories is all instances in which predictions shape sensory experiences. Predictions can suggest the identity of a stimulus or its appearance in time or space. Internal summaries of natural statistics that describe the prevalence of stimuli in natural environments are the most likely driving predictions of stimulus identity [1–3]. Predictions about the time and space of stimulus appearance can be derived from internal or external cues. Predictability of stimulus occurrence is almost perfect when we produce them with our own actions. An abundance of studies has shown that our brains treat self-produced sensations differently than those externally produced. All-day phenomena like the inability to tickle ourselves or the question of why our own voice sounds different to ourselves testify to the impact of predicting the sensory consequences of our own actions [4–8].

**Figure 1.**
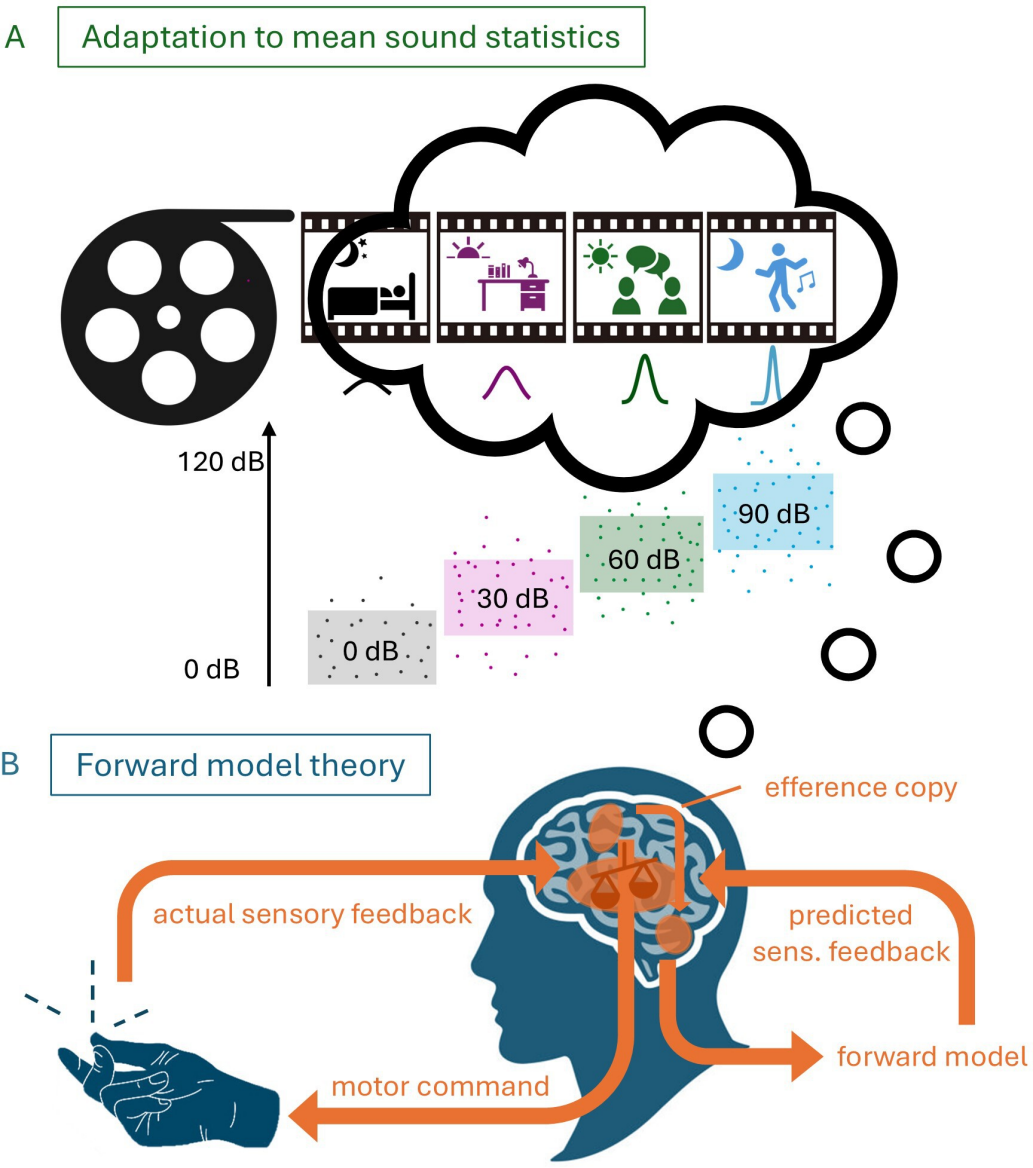
Schematic of adaptation to the mean and the forward model. (A) Different auditory contexts a person has been exposed to over 1 day. According to our theory, this will shape the perception of upcoming stimuli. Coloured boxes show dynamic hearing range that adjusts according to specific contexts. (B) Forward model theory demonstrates the assumed necessity of an efference copy to cause sensory attenuation effects. According to this theory, only self-produced actions can lead to a match between actual and predicted sensory (sens.) feedback.

The favourite account of sensory attenuation contains a forward model architecture whose aim it is to reduce the perceived intensity of self-produced stimulation when predicted and actual sensations match [5,9] (figure 1). The forward model is built up by an efference copy to arrive at a prediction about the upcoming sensory stimulation. Three types of findings currently challenge the forward model as an explanation of sensory attenuation.

First, the prediction of stimulus identity might be irrelevant for attenuation to occur. To attenuate only the self-produced stimulation from the sound spectrum, the prediction must entail its unique features, which in the auditory case is loudness or pitch. Unlike in self-touch, where the execution of a motor plan to touch one’s own body necessarily leads to a tactile sensation on that part of the body, pressing a button does not regularly result in the presentation of a sound. Without training, a connection between the button press and the sound cannot be established. Dogge *et al.* [10] trained subjects to learn certain key tone associations. However, they found no evidence for identity-specific motor predictions in sensory attenuation, neither on a perceptual nor on a neurophysiological level.

Second, studies have reported not only attenuation of self-produced stimulation but also enhancement. While many studies reported that self-produced sounds appeared quieter [11–15], surprisingly, several recent studies also found the opposite that self-produced sounds were judged as louder [11,12,15–17]. This seeming paradox of contradictory evidence needs to be resolved to understand how self-produced sounds are processed by the brain. A hint in this direction was provided by Reznik *et al.* [12], who found attenuation and enhancement in the same study. They reported that self-produced sounds of 75 dB appeared attenuated, whereas sounds close to the individual acoustic threshold appeared enhanced.

Third, some studies have shown that attenuation occurs not only for actively produced but also for passively observed or externally generated stimuli [18,19]. This suggests that the temporal prediction of an event might be sufficient to induce sensory attenuation. However, the literature remains inconclusive about the necessity of an active self-generation compared with equally predictable stimuli. For example, Lange [20] and Klaffehn *et al.* [21] reported attenuation only for self-generated actions compared with passively generated stimuli, while Han *et al.* [18,19] demonstrated that sensory attenuation can also occur in the absence of movement. Supporting this view, Dogge *et al.* [9] argued that predictive mechanisms underlying attenuation do not necessarily depend on self-generation and may go beyond it. In this paper, we sought to study the differences between self-generated and externally generated but temporally predictable stimuli.

An alternative approach to sensory attenuation and enhancement is Bayesian inference. Whereas the forward model operates with concrete sensory predictions based on self-generated stimuli, Bayesian theories rely more on probabilistic inferences about sensory states. In Bayesian perception, uncertain current input is interpreted relative to prior assumptions. Bayesian models suggest that current sensory evidence is combined with prior knowledge about the stimulation, thus enhancing or attenuating the perceived intensity of predicted stimulation [22,23]. By contrast, the forward model cannot explain sensory enhancement [22,24,25].

In a pilot study, originally designed to investigate differences between self- and externally generated stimuli under conditions of predictability and unpredictability, we unexpectedly found enhancement effects for self-generated sounds as well. Notably, we used a sound level of around 49 dB [26], whereas studies reporting attenuation effects often presented sound levels between 60 and 75 dB [12,20]. The parallel evidence for sensory attenuation and for sensory enhancement might indicate that both have a common, yet overlooked, cause. In the present study, we hypothesized that the concurrent existence of attenuation for loud and enhancement for quiet tones [12] might be the signature of a regression towards an average sound level, consistent with the statistical phenomenon known as regression to the mean, where extreme values tend to move closer to the mean. Regression to the mean effects have been observed, for instance in time reproduction tasks, suggesting that the brain takes into account knowledge about temporal uncertainty to adapt internal timing mechanisms to the temporal statistics of the environment [27,28].

Regression effects in loudness perception might be connected to the dynamic hearing shift that adapts the internal sound range to the current acoustic context. Neural populations coding sound level have been shown to use adaptation in order to retain efficiency in animals [1,29–36] and in humans [37,38]. The challenge in loudness discrimination lies in the huge range over which the brain must remain operative. Humans can discriminate sound levels across a 120 decibel (dB) range although neural population codes in the auditory nerve only have a dynamic range between 30 and 40 dB. To cover the entire 120 dB, the dynamic range must be constantly shifted to the current context, i.e. the current sound level statistics [29,39]. Trying to understand someone whispering will produce a different sound level adaptation than when listening to loud music. Different auditory backgrounds can shape your current perception (figure 1A). Sound level adaptation has been investigated in single-cell recordings, and it was found that neurons adjust their responses to the mean, variance and more complex statistics of sound level distributions [29,30]. The increase in coding accuracy near the most commonly occurring sound level should lead to an attractive bias for sound levels outside the adapted dynamic range. The average sound level in all-day human activities is about 50−60 dB. An attractive bias towards this average would lead behaviourally to an attenuation of louder tones and an enhancement for quieter tones. The time course of adaptation is extremely fast with a time constant across neurons of 160 ms [30]. It has recently been reported that neurons in the central thalamus react to temporally predictable stimuli with a decrement of sensory gain corresponding to mean sound level adaptation [40].

Here, we investigated whether sensory enhancement and attenuation result from an adaptation to the average sound level. In this view, quiet tones should result in an enhanced loudness, while louder tones should lead to attenuation. We assumed that predictability of the sound presentation would shift the neural population into adaptation to the mean sound level, resulting in a sensory central tendency. Based on the findings of Reznik *et al.* [12] and our own pilot study [26], we expected a regression to the mean pattern. Producing a tone actively allows perfect temporal predictability about its occurrence. However, we also tested whether passively observed cues indicating tone appearance would affect regression to the mean sound level equally. If the occurrence of tones is as temporally predictable as that of self-generated ones, we would expect to find a similar regression tendency pattern, suggesting that the presence of an efference copy may not be necessary.

## 2. Methods

### (a) Materials and procedures for experiment 1

In total, 110 out of 125 tested participants (*M*_age_ = 22.01 years, s.d. = 3.55 years, 95 female) were analysed in experiment 1. Fifteen participants who provided incorrect responses in more than 25% of the trials with the loudest or quietest reference level were excluded from the analysis. The sample size was chosen to be the same as in a piloting study [26], which was *n* = 55 per group. Participants were randomly and equiprobably assigned to one of two groups. All of them were seated in front of a Dell P2419H Screen with a refresh rate of 60 Hz, that was placed 57 cm away from them. The experiment was built and run in MATLAB [41] using the Psychophysics Toolbox v.3 [42–44]. Used headphones were JBL Tune 500 or Speedlink Garon that were connected via cable to the computer. Physical sound levels of the headphones were measured by a sound level metre (B&K 2250 by Brüel and Kjær). Every used tone was presented for 300 ms with a frequency of 1 kHz. The keyboard was placed 30 cm away from the table edge. After giving their informed consent, the participants received a pseudomized code and participation number.

**Figure 2.**
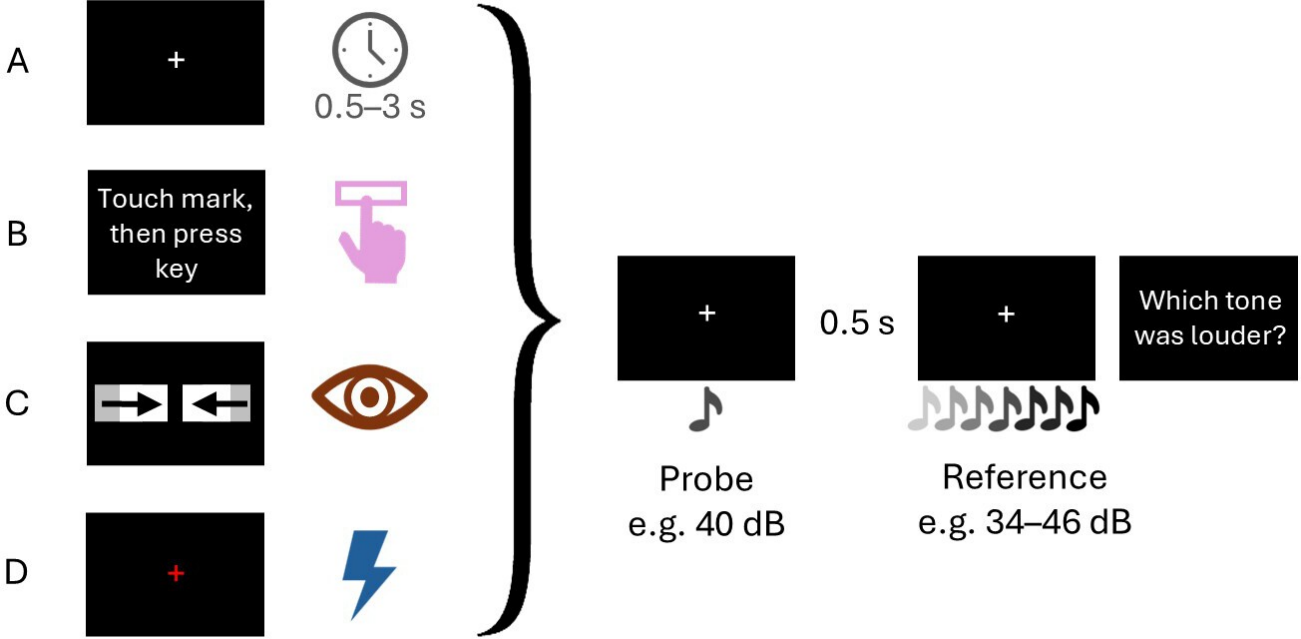
Illustration of experimental trials for experiments 1 and 2. (A) Random interval in the baseline until the appearance of the first tone (experiment 1). (B) Participants triggered the probe tone by pressing a key (experiment 1). (C) The probe tone appeared when two moving bars made contact (experiment 2). (D) A flashing fixation cross served as a cue for the appearance of the probe tone 700 ms later for a duration of 300 ms (experiment 2).

In all tested groups and volume conditions, participants were instructed to keep their gaze directed to a white fixation cross (1° × 1°), presented in the screen centre. In the baseline group, participants were asked to rate which of two presented tones appeared louder. After trial start, the first, i.e. the probe tone, appeared after a random interval between 500 and 3000 ms, a procedure similarly employed in a recent study by Fritz *et al.* [45]. The second, i.e. the comparison tone, was presented 500 ms later. Probe tones were presented in one out of five possible sound volumes (40, 50, 60, 70 or 80 dB) per trial. Every probe volume was presented block-wise but in a randomized order for every participant. For each of the five probe volumes, the respective comparison tone was presented in one out of seven possible sound volumes, which were symmetrically centred around the probe sound volume with a step size of 2 dB. In the 40 dB condition, the comparison tone could have 34, 36, 38, 40, 42, 44 or 46 dB. Each comparison tone was repeated 10 times, resulting in a total of 350 trials per session. After the presentation of the comparison tone, participants responded by pressing the left or the right arrow button. In the active group, participants first touched a mark on the table edge before moving to the keyboard to press the space bar and generate the probe tone. The rest of the trial remained identical to the baseline group. Figure 2A,B illustrates one exemplary trial for both groups.

### (b) Data analysis

For every probe tone, responses for each comparison were averaged within every participant. To these data, a cumulative Gaussian function was fitted. The mean of the psychometric functions was used as the point of subjective equality (PSE) between the two presented tones and served as an index of perceived loudness. For inferential statistics, we planned to analyse the single-subject PSEs by repeated-measures ANOVAs. Raw data files and all used scripts are available at Open Science Framework [26].

## 3. Results of experiment 1: dependence of sensory attenuation on stimulus volume

**Figure 3.**
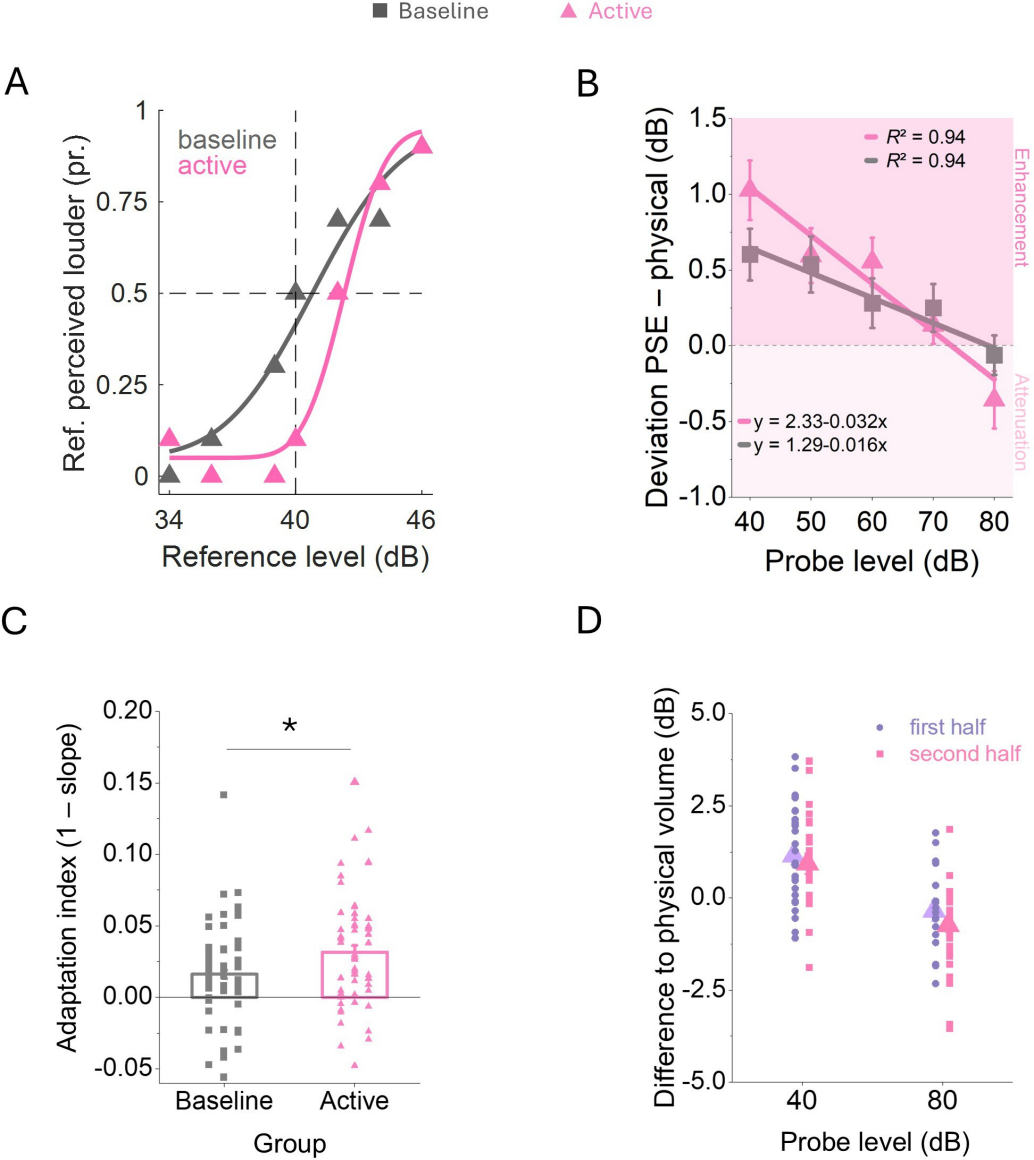
Results for experiment 1: attenuated versus enhanced perception (active). (A) Psychometric functions of one representative participant each. (B) Average loudness estimates shown as the difference to the physical sound level for the baseline (shown in grey) and the active group (shown in pink). Solid lines represent linear functions that best fitted the data. (C) Single subject slopes of the linear fits as the adaptation index for the baseline and the active group. Bars show the mean slopes. (D) Single-subject differences from physical volume for 40 and 80 dB probe levels, separated for the first and second halves of the experiment. Triangles indicate the mean. In all panels, error bars represent s.e.m. *p < 0.05

Responses were analysed as psychometric functions to measure the PSE for every participant (figure 3A). A repeated-measures ANOVA revealed a main effect of presented sound level (F_4,432_ = 24.02, *p* < 0.001, η² = 0.09) and an interaction of sound level with the between-subject factor group (F_4,432_ = 4.80, *p* < .001, η² = 0.02). Figure 3B shows the average perceived loudness as a function of the probe sound level for data measured in the baseline (shown in grey) and in the active group (shown in pink). Baseline data show a systematic negative relationship between loudness estimates and probe level. The quieter the probe tones, the more subjects overestimated them as indicated by the negative slope of the linear fit function. Slopes were used as an index to quantify the strength of the regression. In the active group, we found a negative slope between perceived loudness and probe level that was twice as steep compared to the baseline group. Generally, participants overestimated quiet and underestimated loud tones, a response pattern best described as an adaptation to the average sound level (figure 3C). The average adaptation index of the active group was significantly higher than baseline, *t*(108) = −2.30, *p* = 0.024, *d* = −0.44, s.e. = 0.20.

We then wondered whether the observed regression to the mean reflected the acoustical context that subjects experienced before participating in the experiment or if it was driven by the stimulus distribution itself. To this end, we chose the minimal (40 dB) and the maximal (80 dB) intensities for an exploratory analysis from our probe sound distribution and selected all participants for whom the corresponding blocks of trials were presented either in the first or in the second half of the experiment. When presented in the second half of the experiment, the minimal intensity had been preceded only by louder tones and the maximal intensity only by quieter tones. The selective sampling should have skewed the internal sound distribution, thus amplifying adaptation. Indeed, we found a significant difference for the minimal and maximal probe intensities being presented in the first or second half of the experiment (repeated-measures ANOVA, F_1,104_ = 6.05, *p* = 0.016, η² = 0.06, figure 3D). These results indicate that adaptation to the mean sound level updates quickly enough to incorporate the sound statistics of the experimental session.

## 4. Methods of experiment 2

Next, we investigated why adaptation to the mean sound level increased in the active group in experiment 1. We assumed that actively producing a tone would allow a precise prediction about its temporal occurrence. If temporal prediction led to a shift in the neuron population activity towards the expected volume, then a mere temporal cue should suffice to increase adaptation strength. Here, we aimed to test whether the same regression appears also for passively generated sounds with different levels of predictability. In total, 110 out of 123 tested participants (*M*_age_ = 21.35, s.d. = 2.93, 94 female) were analysed in experiment 2. Thirteen participants who provided incorrect responses in more than 25% of the trials with the loudest or quietest reference level were excluded from the analysis. In the first passive group, a continuous cue was introduced: two white bars (size: 5.3° × 1°) were shown that moved towards each other starting at −6.75° and +6.75° and made contact in the screen centre after 70 frames. Here, the probe tone was presented simultaneously when the bars made contact. In the second passive group (flashed-cue passive group), 700 ms after trial start the fixation cross flashed red for 300 ms. The colour change served as a cue for the tone, which always appeared 300 ms later. Used materials and programmes remained the same as in experiment 1. Figure 2C,D illustrates one exemplary trial for each group.

## 5. Results of experiment 2: sensory consequences for passively generated, cued stimuli

**Figure 4.**
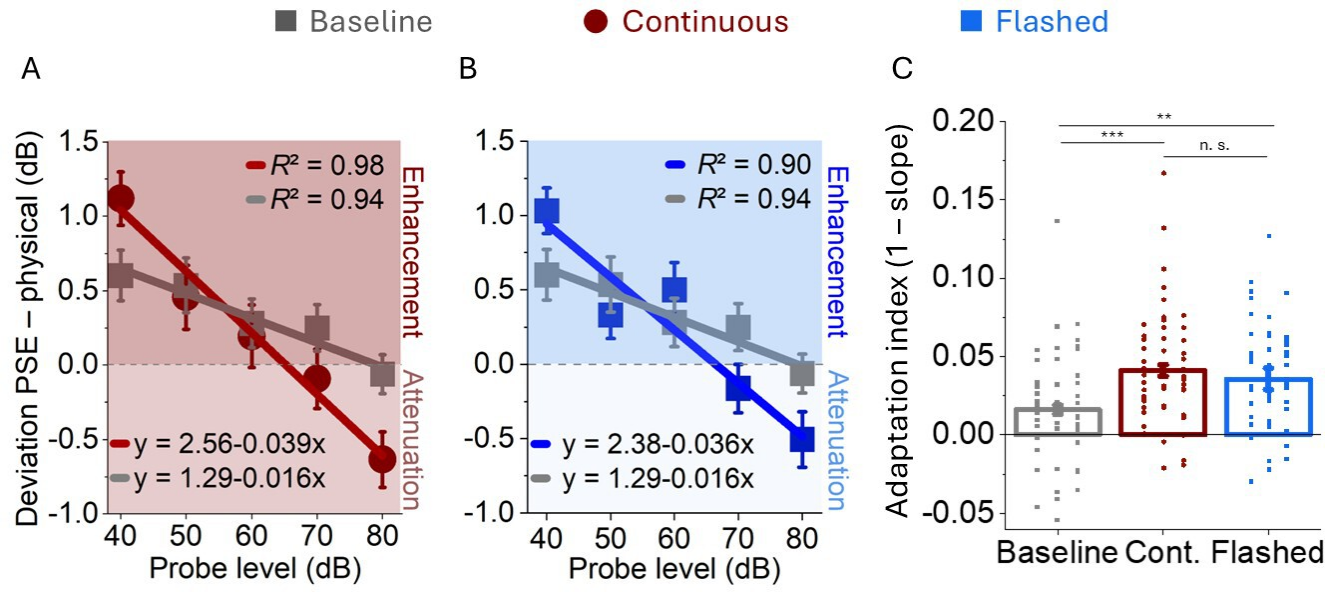
Results for experiment 2: attenuated versus enhanced perception (passive). (A,B) Average loudness estimates shown as the difference to the physical sound level for the continuous (shown in brown), (A), and the flashed-cue group (shown in blue), (B) compared with the baseline (shown in grey). Solid lines represent linear functions that best fitted the data. (C) Single subject slopes of the linear fits as an adaptation index for the baseline, the continuous and the flashed conditions. In all panels, error bars represent s.e.m. **p < 0.01, ***p < 0.001, n. s. not significant.

A repeated-measures ANOVA based on the PSE differences of the continuous, flashed and baseline groups revealed a main effect of presented sound level (F_4,648_ = 47.36, *p* < 0.001, η² = 0.10) and an interaction of sound level with the between-subject factor group (F_8,648_ = 3.69, *p* < 0.001, η² = 0.02) (figure 4A,B). The quieter the probe tones, the more subjects overestimated them as indicated by the negative slope of the linear fit function. An ANOVA of the slopes as the adaptation index for the same three groups revealed a significant effect of group (F_2,162_ = 7.76, *p* < 0.001, η*²* = 0.09, figure 4C). A follow-up Bayesian *t*‐test comparing the different groups from experiments 1 and 2 provided moderate evidence in favour of the null hypothesis of no differences between the groups over the alternative, with a Bayes factor of BF₁₀ = 0.32 (error % = 0.02) between the active and the continuous group, as well as between the active and the flashed group (BF₁₀ = 0.23, error % = 0.03) and between the two passive groups (BF₁₀ = 0.23, error % = 0.03). The analysis was performed using a Bayesian *t*‐test with a two-sided Cauchy prior distribution centred at zero with a scale parameter *r* = 0.707, as implemented as default in JASP v.0.18.1.0 [46]. Descriptive statistics of mean PSE differences for all groups and sound levels can be found in electronic supplementary material, table S1.

## 6. Methods of experiment 3

In experiment 3, we collected data of 118 participants and included 110 in the final sample (*M*_age_ = 22.16, s.d. = 5.67, 88 female). The desired sample size was chosen to match the size of each group from experiments 1 and 2. Eight participants who provided incorrect responses on more than 25% of the trials with the loudest or quietest reference level were excluded from the analysis. Materials and programmes were identical to experiments 1 and 2.

One group of 56 participants was assigned to a quiet context session, and the other group of 54 participants to a loud context session. The quiet context session started with a baseline condition of 70 trials in which the probe tone was presented with a volume of 80 dB. The comparison tone was presented in one out of seven possible sound volumes (symmetrically centred around the probe sound volume with a step size of 3 dB). The trial structure was identical to that of the baseline group in experiment 1 (figure 2A). Then, a second baseline of 70 trials was measured for a 40 dB probe tone. After the two baseline conditions, another 70 trials were performed in the volume of the desired context (40 dB). During the experimental phase, in the majority of trials (80%, 280 trials), the probe tone was presented with a volume of 40 dB. In the remaining 20% (70 trials), a probe tone with 80 dB was presented. Loud context sessions started with a baseline condition in which a 40 dB probe tone was presented. In the second baseline, an 80 dB probe tone was presented. Since the desired context was 80 dB, participants performed another 70 adaptation trials in that volume. In the loud context condition, in 80% of all trials an 80 dB probe tone and in 20% a 40 dB probe tone were measured. Trial structure during the experimental phase was identical to the one in the active group in experiment 1 (figure 2B), since we only used self-generated trials for experiment 3.

## 7. Results of experiment 3: adaptation to the mean sound statistics

**Figure 5.**
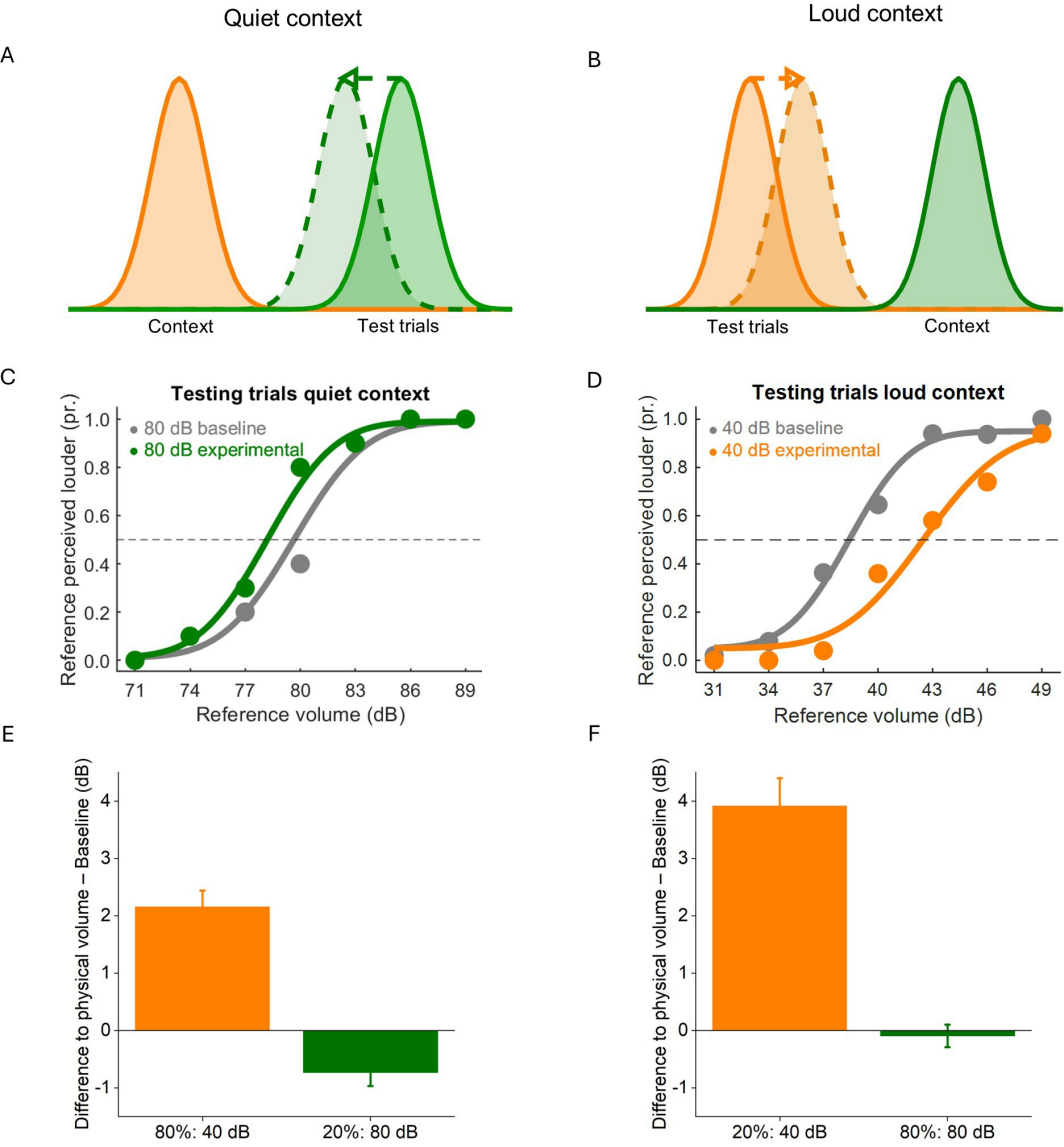
Results for experiment 3: adjustability of the mean sound statistics. (A,B) Schematic of hypotheses. (A) When the context consists of quiet tones (40 dB, shown in orange), loud tones presented in rare test trials (80 dB, shown in green) should shift and appear quieter. (B) When the context consists of loud tones (80 dB, shown in green), quiet tones presented in rare test trials (40 dB, shown in orange) should shift and appear louder. (C,D) Representative psychometric functions of two participants in the quiet and loud context conditions. (E,F) Bar plots showing the mean deviation from physical volume for the majority and minority of presented trials for quiet and loud contexts. In all panels, error bars represent s.e.m.

In experiment 3, we created artificial sound contexts by presenting in 80% of all trials either loud (80 dB) or quiet tones (40 dB). If the context serves as prior and leads to adaptation to the mean sound level, perceived loudness in the test trials should shift towards the context sound level (see figure 5A,B). We expected that when presenting a loud tone to a participant who has been exposed to a quiet context, it should be perceived as more quiet (figure 5A), while the presentation of a quiet tone to a participant who has been exposed to a loud context should lead to an effect of enhancement (figure 5B).

When loud tones were presented in the loud context, they were perceived as intense as in baseline trials (figure 5F). However, the intensity of loud tones intermixed into a quiet context appeared attenuated compared with the baseline (figure 5E). A repeated-measures ANOVA revealed a significant effect of sound level (40 versus 80 dB, F_1,108_ = 126.21, *p* < 0.001, η² = 0.14), as well as a significant interaction with the factor context (F_1,108_ = 17.24, *p* < 0.001, η² = 0.02), a significant difference to the baseline (F_1,108_ = 58.93, *p* < 0.001, η² = 0.06) and again an interaction with context (F_1,108_ = 12.30, *p* < 0.001, η² = 0.01).

Figure 5C shows a slight shift in psychometric functions of one representative participant for 80 dB trials that were presented in the quiet context compared with the baseline. Figure 5D shows the corresponding counterpart of 40 dB trials that were presented as rare trials in a loud context, which causes an enhancing shift compared to the baseline. When presented in the quiet context, the loudness of quiet tones was overestimated less (figure 5E). By contrast, when interspersed into the loud context trials, the loudness of quiet tones was massively overestimated by an average 4 dB (figure 5F).Descriptive statistics can be found in electronic supplementary material, table S2.

## 8. Discussion

Our results show that loudness estimates of self-produced or passively observed but cued tones regress to the average sound level. Observers overestimate the volume of quiet and underestimate the volume of loud tones. In our experiments, regression could already be seen in the baseline, but its magnitude increased in the experimental conditions. We will discuss first the implications of our study for the sensory attenuation/enhancement effects and will then elaborate on the regression to the mean sound level.

Our results have strong implications for the phenomenon called sensory attenuation. They resolve the apparent paradox of concurrent reports showing either attenuation or enhancement in comparable experimental set-ups [11–15,17]. The effects of regression to the mean imply that attenuation and enhancement are two sides of one coin. Both result from a subjective loudness shift to the mean sound level. Attenuation and enhancement are consequences of this shift, emerging when sounds quieter or louder than the average are presented.

In previous research, most studies found sensory attenuation rather than enhancement [47]. Consistent with the regression explanation, the effect was primarily investigated in electroencephalogram studies that used high sound levels (>70 dB) to obtain stronger neural signals. Focusing on the potential functional benefit of sensory attenuation, the forward model provided a mechanistic explanation about the suppression of self-produced sensory stimulation. However, if attenuation and enhancement result from a shift towards the central tendency of sensory stimulation, then the search for dedicated mechanisms to explain these effects should be replaced by a search for what explains the regression.

The forward model account of sensory attenuation has been criticized recently [9,23,48]. Unlike self-produced tactile sensations, it is unclear why sounds should be attenuated in the first place. A piano player’s brain would not be well advised to suppress the played sounds. The forward model idea stands on three legs, which are the stimulus identity prediction, the suppression if the prediction is matched by the sensation and finally, the efference copy as an input. Our results indicate that none of the legs of the stool are holding up, as we will now discuss.

First, effects of identity prediction could not be confirmed in a behavioural study [10]. Second, the forward model account is inconsistent with enhancement effects and has been criticized as insufficient [9,23,49]. In our data, sensory enhancement was dominant across a much wider range of probe sound levels than attenuation, which we found only for the loudest sound level. Third, by definition, an efference copy will only be generated in active tasks. In experiment 2, we only used passive cues to mark the appearance of the first tone. We found moderate Bayesian evidence for the hypothesis that the active and passive groups from experiments 1 and 2 do not differ from each other concerning their strength of regression. The regression to the mean sound level seems to be a general effect that does not depend on the source of generation. Conversely, Arikan *et al.* [50] investigated differences between active and passive movements and their influences on the perception of vibrating stimuli. Even though sensory attenuation was found for both active and passive movements, the active condition still led to larger suppression effects. However, it is not yet clear whether auditory and tactile attenuation effects are comparable or related. In our auditory study, all experimental groups affected the regression equally. This result opens up the possibility that an efference copy is not necessary to produce perceptual changes of self-produced stimuli. The temporal prediction of stimulus occurrence might be sufficient. On the one hand, we cannot exclude the possibility that in active and passive trials separate mechanisms are at play. On the other hand, if temporal prediction produces the regression effects, it is questionable to postulate an additional mechanism that arrives at the similar outcome.

In regression to the mean effects, the perceived intensity of a stimulus results from a weighted average of the actual stimulation and an anchor value [51]. The anchor might consist of a central tendency measure that is derived either from the experimental stimulus distribution or from long-term natural statistics. In real life, the loudness frequency of acoustic stimulation centres around 60 dB, i.e. the human communication sound level. A common research question is which number of samples is necessary to learn or update the sound distribution.

In our experiments 1 and 2, we presented stimuli block-wise with 70 trials per block. Such a presentation order is usually chosen to avoid a regression to the mean stimulus distribution [27]. Yet, we still found regression to the mean effects. It is therefore likely that our data reflect a long-term representation of acoustic statistics that is not yet overwritten by the stimuli presented in the experiment. In experiment 3, we presented an identical sound level, either 40 or 80 dB, over 80% of all trials (i.e. in 350 trials) and found differences when comparing both conditions. Placed within a loud context, quiet tones were judged to be much louder than they actually were. We also found that loud tones presented in a loud sound context were no longer attenuated. These differences suggest that between 70 and 350 trials are necessary to update the internal representation of the sound statistics.

We found a surprisingly strong enhancement effect in the loud context conditions. Given that the increase in enhancement is much stronger than the decrease in attenuation, an additional factor might come into play. Beyvers *et al.* [52] reported more pronounced tactile suppression for strong compared with weak tactile feedback. They suggested that stimuli with weak intensities are upregulated by the perceptual systems to not miss them. In the loud context, quiet tones are presented in only 20% of all trials. It might be that subjects paid additional attention to the tones in the loud context in order not to miss the quiet tones.

Regression to the mean effects might be a signature of an inference about the stimulus intensity. The task of the participants is to judge the intensity of a probe sound that has to be briefly stored in memory to be compared against a reference stimulus. To infer the loudness of the probe sound, the inference process might involve an internal sound representation [53,54]. Consistent with this idea, in experiment 1, regression was already observed in the baseline. This is in line with conclusions from Kiepe *et al.* [53] stating that sensory attenuation is rather based on learnt associations than on generated behaviour [9,55–57]. In a constant process, we use prior information to create predictions about following sensory input. However, regression strength increased when the tone was either self-produced or temporally cued. Temporal predictability of stimulus occurrence might allow a stronger involvement of the internal sound statistic representation to create predictions.

A neural process that supports loudness estimates is the shift of the dynamic sound range. Although we can hear sounds between 0 and 120 dB, precise loudness judgements can only be accomplished with a much smaller range. A dynamic sound range adaptively shifts to the current sound context in order to match this window to the current stimulation. We speculate that the dynamic sound range might shift to the mean of the internal representation. If the mean is located between 60 and 70 dB, our observed regression effects might be the consequence. Adaptation to the mean sound level has been observed in neural responses in the auditory midbrain [1,29,31–35]. Neural adaptation followed an increase in the mean sound level with an average time constant across neurons of 160 ms [30] and might be induced by neurons in the central thalamus that predict temporal stimulus regularities [40]. Since dynamic shifts in auditory neurons are already well documented [30,40], it is likely that this mechanism affects the behavioural perception of predictable tones. Our data show that adaptation to the mean sound level explains why the self-produced sounds appear different to us. Temporal predictions about stimulus occurrence, which are internally available during action planning, might shift the neuron population into adaptation to the mean sound statistics. In humans, Herrmann *et al.* [38] found that neural-response sensitivity is influenced by specific sound-level contexts, and neurons are most sensitive to sound levels that differ from the established and thus predictable context. This might be a neuronal explanation for the strong enhancement effects in the perception of quiet trials in the loud context condition. In our data, the context effect was markedly stronger for the enhancement of quiet tones than for the attenuation of loud tones. Although this link is rather speculative, it was found that neurons adapt faster to increases of loudness than to decreases [30]. If this asymmetry is related to the data of our second experiment, it would explain why quiet tones adapted more strongly to loud tones than *vice versa*.

In conclusion, our data suggest that sensory attenuation and enhancement of self-produced and passively observed, cued sounds stem from a regression to the average sound level. These results challenge the forward model account of sensory attenuation on its main premises.

### (a) Limitations

All experiments were conducted in a laboratory setting. In the tactile domain, we can anticipate how self-touch will feel. However, in our experiments, participants pressed a key that triggered a tone. Before the first trials, they had no prior knowledge of how this tone would sound, nor of its volume or frequency. The association between a key press and a tone is rather artificial and may require time to be learnt.

## Data Availability

Raw data files, all used scripts and supplementary materials are available at Open Science Framework [58]. Supplementary material is available online [59].
